# Fungal cell wall: An underexploited target for antifungal therapies

**DOI:** 10.1371/journal.ppat.1009470

**Published:** 2021-04-22

**Authors:** Chibuike Ibe, Carol A. Munro

**Affiliations:** 1 Department of Microbiology, Abia State University, Uturu, Abia State, Nigeria; 2 Aberdeen Fungal Group, Institute of Medical Sciences, University of Aberdeen, Aberdeen United Kingdom; University of Maryland, Baltimore, UNITED STATES

## Cell wall architecture

**Fig 1:**
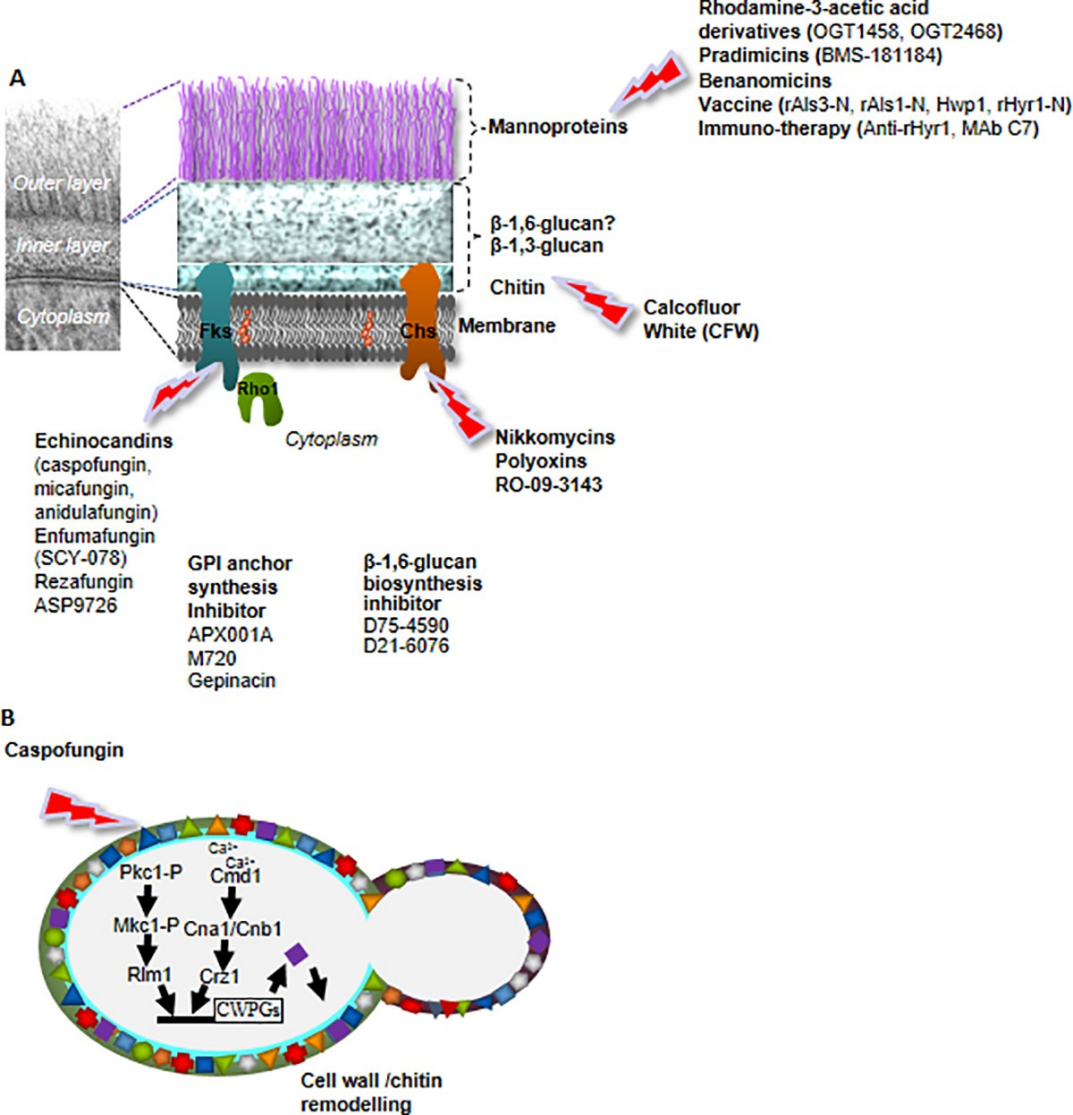
**A model illustrating cell wall architecture and antifungal agents targeting specific components of the cell wall (A).** Several antifungal agents are available that target the cell wall, but only the echinocandins have been licenced for the treatment of fungal infections; research into β-1,6-glucan inhibitor has been relatively unsuccessful. **Cell wall integrity pathway leading to caspofungin drug tolerance (B).** The Pkc pathway is activated in response to damage to the cell wall caused by inhibition of β-1,3-glucan synthesis by echinocandins. The signal that cell wall is weakened is transduced by membrane-bound mechanosensors such as members of the Wsc family, to activate Rho1. Rho1 activates Pkc1 which in turn activates the downstream MAP kinase cascade leading to the phosphorylation of Mkc1. A number of transcription factors are then activated including Cas5 and Rlm1 that switch on the expression of genes involved in cell wall construction and remodelling. The calcium signalling pathway is involved in cell wall salvage response. The pathway is activated by Ca^2+^ that enters the cell through membrane-localised channels or is released from intracellular stores. Ca^2+^ binds to and activates calmodulin (Cmd1). Cmd1 activates the phosphatase calcineurin, which is made up of 2 subunits Cna1 and Cnb1. Calcineurin dephosphorylates the transcription factor Crz1. Crz1 then moves into the nucleus and induces the expression of genes through binding to CDREs within their promoter sequences. The calcium signalling pathway regulates *CHS* genes. CDREs, calcium-dependent response elements; CWPGs, cell wall protein genes; GPI, glycosylphosphatidylinositol.

The cell wall of the major fungal pathogen *Candida albicans* consists of an outer layer that is made up of highly mannosylated proteins. The proteins are attached to the inner layer of the cell wall and appear perpendicular to the cell surface forming a fibrillar protein coat (**Fig 1A**). The polysaccharide rich inner layer is mainly made up of β-1,6- and β-1,3-glucans and chitin while β-1,3-glucans and chitin are the structural polysaccharides in the cell wall.

The development of antifungal therapies that target the cell wall has received great attention (**Fig 1A**) but with only limited success. The only cell wall-targeting antifungal agents, licenced for human infections, are echinocandins.

## Which cell wall polysaccharide is the most attractive antifungal target?

### Chitin

Chitin is a linear homopolymer of β-1,4-linked *N*-acetylglucosamine (GlcNAc) and is essential for cell viability. Chitin is synthesised by a family of membrane-localised chitin synthase enzymes (Chs1 to 3, and Chs8 in *C*. *albicans*) and Chs1 is essential [1]. Polyoxins and nikkomycins are potent inhibitors of Chs enzyme that compete with Chs substrate UDP-GlcNAc for Chs binding, due to their structural similarity but have limited effects on whole cells. Nikkomycin Z is effective against *Coccidioides* species causing respiratory infections. The drug reduced the fungal pulmonary burdens by 6-log^40^ 2 days post infection, but clinical trials were terminated due to lack of finance [1,2].

The enzymes involved in chitin synthesis have specialised functions but can be functionally redundant under specific conditions. In addition, subtle differences in protein structure between Chs family members has complicated the development of efficient chitin synthase inhibitors. For example, a Chs1-specific inhibitor, RO-09-3143, blocked septum formation by Chs1 and arrested cell growth, but inhibition of Chs1 was only lethal in *chs2Δ* deletion mutant suggesting functional redundancy [3]. Other chitin synthase inhibitors such as the 3-substituted amino-4-hydroxycoumarin derivatives have also been found to have antifungal activity [4], but none has made it to the clinic.

### β-1,3-glucan

β-1,3-glucan is a polymer of glucose units and can exist in a stable triple helical structure which gives it some degree of elasticity and tensile strength making it the main structural polysaccharide in the cell wall of most fungi [5]. β-1,3-glucan has been the most attractive antifungal target due to its central structural role in the cell wall. β-1,3-glucan is synthesised by essential β-1,3-glucan synthase enzymes composed of an integral membrane protein catalytic subunit, Fks, and a regulatory subunit, Rho1 (**Fig 1A**). The echinocandins (caspofungin, micafungin, and anidulafungin) are a class of antifungal agents that noncompetitively inhibit β-1,3-glucan synthesis through inhibiting Fks [6]. They are fungicidal against *Candida* species [7]. Resistance to the echinocandins in *Candida* species is due to *FKS1* hotspot mutations, and tolerance occurs due to cell wall remodelling and compensatory up-regulation of chitin synthesis. Recently, other β-1,3-glucan inhibitors such as enfumifungin have been identified. The lead molecule SCY-078 (ibrexafungerp) is an orally active synthetic derivative of enfumifungin with in vitro antifungal activity. In vivo, ibrexafungerp is extensively distributed in the tissue and is active against *Candida* and *Aspergillus* species [8]. Drug resistance to ibrexafungerp was mapped to *FKS1*, although strains resistant to the echinocandins do not display cross-resistance to ibrexafungerp, thus emphasising different mechanisms of action for both agents [9–12]. A Phase I clinical trial of the drug showed mild-to-moderate inflammation-related thrombotic events in healthy volunteers; oral and intravenous formulations set for Phase II clinical trial in the third quarter of 2018 have been completed, while a Phase III clinical trial to evaluate the efficacy of oral ibrexafungerp on recurrent vulvovaginal candidiasis is ongoing and estimated to be completed in September 2021.

Other β-1,3-glucan synthesis inhibitors under development include rezafungin (CD101), a novel echinocandin [13], and piperazinyl-pyridazinone [14]. Rezafungin is a structural analogue of anidulafungin with in vivo activity against *Candida* and *Aspergillus* species. Rezafungin is currently in Phase III clinical trial. The piperazinyl-pyridazinones have in vitro activity against *Candida* and *Aspergillus* species as well as other fungi and have potency in vivo against *Candida glabrata*. Piperazinyl-pyridazinone is effective against echinocandin-resistant *C*. *albicans* probably because it binds to a different region of Fks1 from echinocandins [14]. Significant success has been seen in developing β-1,3-glucan synthesis inhibitors partly because β-1,3-glucan is synthesised by a single synthase.

### β-1,6-glucan

β-1,6-glucan plays a central role in cell wall organisation and structure by linking cell wall proteins (CWPs) to the cell wall matrix [15]. Several genes such as *KRE9*, *KRE6*, and *KRE5* have been associated with β-1,6-glucan synthesis in *S*. *cerevisiae* and *C*. *albicans* [16]. The synthesis of β-1,6-glucan has been proposed as a promising drug target strengthened by the fact that *Ca*Kre9 is essential. The unavailability of a specific protein whose catalytic activity is directly linked to the synthesis of β-1,6-glucan has made the development of inhibitors difficult despite its importance in cell wall organisation. Pyridobenzimidazole derivative, D75-4590 was found to target and inhibit Kre6. *C*. *albicans* cells treated with D75-4590 had reduced level of β-1,6-glucan. Other derivatives of pyridobenzimidazole, such as D21-6076, have in vitro and in vivo activities against *C*. *albicans* and *C*. *glabrata*. Synergistic effect has been detected in the interaction of a β-1,6-glucan inhibitor, D11-2040, and caspofungin against *C*. *albicans* [17].

## Are cell wall mannoproteins better antifungal targets than cell wall polysaccharides?

The cell wall mannoproteins are made up of several classes; 88% are glycosylphosphatidylinositol (GPI)-modified proteins. GPI-modified mannoproteins are involved in cell wall synthesis, organisation and remodelling, and virulence. These include proteins with glycosylhydrolase, glycosyltransferase, or transglycosidase activities such as Crh and Phr families that function in the extracellular space to modify, assemble, and crosslink the essential cell wall polymers extruded into the wall space. These enzymes are excellent antifungal targets that act downstream of cell wall salvage pathways and inhibiting their synthesis and localisation to the cell wall could impact on virulence and cell wall biosynthesis significantly. Molecules have been identified which can interfere with the posttranslational modification of cell wall mannoproteins: glycosylation and the synthesis and attachment of the GPI anchor. One is a pyridine-2-amine-based molecule, APX001A, which inhibits the acylation of the inositol ring during GPI anchor synthesis, a step catalysed by Gwt1, an inositol acyltransferase. Deletion of *GWT1* is lethal, or leads to temperature sensitivity and slow growth, depending on strain genetic background. APX001A is effective at low concentrations against *Candida*, *Aspergillus*, *Fusarium*, and *Scedosporium* species. It is also effective against caspofungin-resistant *C*. *albicans*. APX001A had elevated in vitro and in vivo activities against *Candida auris* compared to anidulafungin. Phenoxyacetanilide (gepinacin) can also inhibit Gwt1 and has antifungal activity against diverse yeasts and moulds [18]. M720 is an inhibitor of phosphoethanolamine transferase-I, Mcd4, involved in the GPI anchor synthetic pathway with in vivo activity against *C*. *albicans* [19]. APX001A and M720 have synergistic effect against *C*. *albicans* [19]. Other enzymes involved in the GPI anchor synthesis such as Smp3 have also been proposed as therapeutic targets [20,21]. Rhodamine-3-acetic acid derivatives such as OGT2468 inhibit cell wall mannoprotein synthesis by inhibiting protein glycosylation [22].

Cell wall mannoproteins as potential drug and vaccine targets present a new opportunity to combat fungi infection, though it has gained relatively less attention/success compared to the development of antifungal agents that target β-1,3-glucan synthesis.

## Are cell wall localised virulence factors promising targets for vaccine and immunotherapy development?

There are no licenced vaccines for fungal diseases and most individuals who suffer from systemic mycoses have impaired immunity that makes them susceptible to invasive infections. With impaired immunity, it would be difficult for immune-based therapies to eradicate the infection. This limited effectiveness of antifungal drugs could be improved by developing antibody therapies. The fungal cell wall glycoproteins are involved in adhesion to surfaces, medical devices, and host cells. GPI-modified proteins are required for morphogenesis, virulence, and resistance to macrophages, making them potential candidates for vaccine and immunotherapy development [23].

The main group of adhesins in *C*. *albicans* is the family of 8 agglutinin-like sequence (Als) surface glycoproteins encoded by the *ALS* genes. The Als proteins are potent virulence factors and the recombinant N termini of Als1 and Als3 have been shown to have potential as prophylactics. The rAls1-N vaccine was found to protect mice against oropharyngeal and disseminated candidiasis. The rAls3-N vaccine protects mice against *C*. *albicans* infections by stimulating Th1/Th17 lymphocytes to produce high levels of IFN-γ and IL-17A as well as chemokines. These cytokines enhance the capacity of phagocytes to kill the pathogen. The vaccine has been shown to confer protection to immunocompetent mice from systemic, oropharyngeal, and vaginal candidiasis. Human trials with the rAls3-N vaccine are ongoing [24], and data show that the vaccine is safe and induce rapid and robust immune response [25].

Hwp1 and Hyr1 are outer surface mannoproteins found on germ tubes and hyphal cells of *C*. *albicans*. Recombinant Hyr1 vaccine has been found to protect mice against haematogenously disseminated candidiasis. Anti-rHyr1 antibodies neutralise Hyr1 antigenic effect in vitro and in vivo [26]. Hwp1 has been used in a synthetic glycopeptide vaccine construct, combining β mannan and a peptide epitope, which was found to protect mice against disseminated candidiasis. These vaccine candidates are promising and may gain clinical approval soon.

*C*. *albicans* uses receptor proteins such as Rbt5 on the cell surface to take up nutrients such as iron during infection. Monoclonal antibody C7 was thought to exert candidacidal activity by blocking the iron uptake pathway in *C*. *albicans*, though this effect is reversible by adding iron to the growth medium [27].

Inhibiting or blocking these cell surface virulence factors may be useful in reducing the progress of fungal infections especially with drug-resistant strains. However, the abundance and redundancy as well as compensatory activities in the function of these proteins have stalled rapid development of drugs/vaccines/immunotherapies. Thorough investigation of each cell surface protein may broaden the available protein candidates for the development of prophylactics, immunotherapies, and therapeutics.

## How do cell wall compensatory mechanisms that lead to cell wall remodelling impact on antifungal efficacy?

Echinocandins are used as a first-line drug against invasive candidiasis. Resistance to echinocandins has developed through acquisition of point mutations in *FKS1* or its paralogues. These point mutations are clustered around 2 hotspots mapped to amino acids at positions 641 to 649 (hotspot 1) and 1,345 to 1,365 (hotspot 2). Point mutations that alter echinocandin sensitivity decrease enzyme velocity, V_max_, but not the binding affinity, K_m_, of the drug for Fks enzyme. Serine is frequently substituted with phenylalanine, proline, or tyrosine at position 645. These mutations confer resistance as homozygous and heterozygous alleles to all 3 echinocandins. However, not all *FKS1* mutations are associated with reduced susceptibility to the echinocandins [28]. Recently, the SENTRY surveillance project has examined the prevalence of *FKS1* mutations in echinocandin-resistant isolates and found that the majority of resistant isolates of the major pathogenic *Candida* species tested do not harbour these mutations [29] suggesting that alternative mechanisms including cell wall remodelling (**Fig 1B**) may contribute to drug resistance. β-1,3-glucan and chitin, the 2 cell wall structural polymers reinforce and complement each other in a dynamic process mediated by wall glycoproteins to maintain an intact cell wall. Cell wall remodelling alters the susceptibility of *C*. *albicans* and *Aspergillus fumigatus* to echinocandin drugs. In vitro treatment of *C*. *albicans* with subinhibitory concentrations of caspofungin induces increased chitin synthesis as a compensatory response, through activation of the Pkc, calcineurin (**Fig 1B**), and HOG signalling pathways. Activation of the cell wall damage pathway leads to cell wall/chitin remodelling resulting in a thicker wall with increased chitin content. *C*. *albicans* cells with elevated chitin content in the wall are less susceptible to caspofungin treatment in vitro and in vivo [30]. The observed elevation in cell wall chitin content appears to be an adaptive mechanism to maintain an intact cell wall and not a genetic alteration but remodelling of the cell wall may give the fungus a window of opportunity to acquire the point mutations that subsequently fix echinocandin resistance in the population. Some *C*. *albicans*-resistant clinical isolates have been found to have hotspot mutations in addition to a thicker wall with elevated wall chitin content [31,32]. This has led to the proposal that combination therapies that block both glucan and chitin synthesis may be attractive to abrogate the acquisition of caspofungin tolerance and resistance.

The CWI pathway and activation of chitin synthesis must be overcome in order to reduce the resistance to and increase the effectiveness of β-1,3-glucan-targeted therapies. Several new β-1,3-glucan synthase inhibitors have been discovered. If they are clinically approved, they may share the same fate as the echinocandins, activating compensatory cell wall remodelling activities when used at suboptimal concentrations or due to reduced bioavailability in some host niches. Thus, a clinically approved agent that directly and noncompetitively inhibits the synthesis of chitin may improve the clinical usefulness of β-1,3-glucan inhibitors in terms of spectrum of activity and effectiveness.

The cell wall remains one of the most attractive antifungal targets yet it is a virtually an unexploited area of research compared to the bacterial cell wall in terms of clinically approved wall-targeting drugs. Even with recent successes in understanding cell wall biogenesis, more research is needed if more effective antifungal therapies are to earn clinical approval.
